# Variation in sex allocation plasticity in three closely related flatworm species

**DOI:** 10.1002/ece3.5566

**Published:** 2019-08-16

**Authors:** Pragya Singh, Nikolas Vellnow, Lukas Schärer

**Affiliations:** ^1^ Evolutionary Biology Zoological Institute University of Basel Basel Switzerland; ^2^ Evolutionary Biology Department Bielefeld University Bielefeld Germany

**Keywords:** local mate competition, local sperm competition, self‐fertilization, simultaneous hermaphrodites, sperm morphology, sperm production rate

## Abstract

Sex allocation (SA) theory for simultaneous hermaphrodites predicts an influence of group size on SA. Since group size can vary within an individual's lifetime, this can favor the evolution of phenotypically plastic SA. In an emerging comparative context, we here report on SA plasticity in three closely related *Macrostomum* flatworm species, namely *Macrostomum janickei*, *Macrostomum cliftonensis*, and *Macrostomum mirumnovem*. For each species, we experimentally raised worms in three group sizes (isolated, pairs, and octets) and two enclosure sizes (small and large) in all factorial combinations and studied the effects of these factors on different estimates of SA. In addition, we also evaluated whether isolated worms engage in self‐fertilization. We found that all species have plastic SA, with *M. cliftonensis* being more plastic than the other two species, as assessed by comparing standardized effect sizes of (a) the presence/absence of mating partners and (b) the strength of sexual competition. Moreover, we found that sperm production rate—but not sperm morphology—is plastic in *M. cliftonensis*, and that only *M. mirumnovem* self‐fertilized during our observation period. Our study suggests that both SA and SA plasticity can diverge even between closely related species.

## Graphical abstract

The evolution of sex allocation plasticity is still poorly understood. We report on substantial variation in sex allocation plasticity between three closely related species, suggesting that it can evolve rapidly even between closely related species.

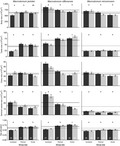

## 1 INTRODUCTION

Sex allocation (SA) theory in simultaneous hermaphrodites (hermaphrodites henceforth) predicts an influence of mating group size on SA, with small mating group sizes favoring a female‐biased SA and larger mating group sizes favoring a more equal SA (Charnov, 1982). This effect of mating group size on SA can be understood in terms of local sperm competition (LSC; Schärer, 2009; Schärer & Pen, 2013), which occurs when related sperm from the same sperm donor (or related sperm donors) compete for access to a partner's eggs. LSC can be thought of as the inverse of sperm competition, which in turn occurs when unrelated sperm compete for access to a partner's eggs (Parker, 1970, 1998). In the presence of high LSC—for example, under monogamy or obligate self‐fertilization—the competing sperm are maximally related to each other, and thus, fitness returns for male investment increase at a strongly diminishing rate (Charlesworth & Charlesworth, 1981; Charnov, 1982; Schärer & Pen, 2013). This happens because any investment into the male function to produce more sperm than are required to fertilize the partner's eggs is a waste of resources, as the donor‐related sperm simply compete among themselves. Under these conditions, a hermaphroditic individual can instead maximize its fitness by reallocating investment from sperm production (male function) to the production of its own eggs (female function), thus favoring a female‐biased SA under monogamy or obligate self‐fertilization (Charlesworth & Charlesworth, 1981; Charnov, 1982; Schärer, 2009; Schärer & Pen, 2013). But as the mating group size increases, leading to more donors contributing sperm to each recipient, an individual's sperm competes more and more with unrelated sperm. Under these conditions, it pays off to invest into the male function to gain more fertilizations, favoring a subsequent shift toward a more equal SA (Charnov, 1982; Schärer, 2009; Schärer & Pen, 2013).

Most SA models predict the effect of mating group size and LSC in evolutionary terms, with SA representing an adaptation to the average mating group size experienced by a species over many generations and thus being modeled as a genetically fixed trait (but see Charnov, 1986). In contrast, most empirical tests of SA theory for hermaphrodites have evaluated these predictions based on the premise that SA can also be plastically adjusted, in response to environmental conditions experienced by the organism, either during their ontogeny (e.g., Janicke et al., 2013; Janicke, Sandner, Ramm, Vizoso, & Schärer, 2016; Schärer & Ladurner, 2003; Schärer & Wedekind, 2001; Tan, Govedich, & Burd, 2004) or during adulthood (e.g., Brauer, Schärer, & Michiels, 2007; Hart, Svoboda, & Cortez, 2011; Hoch & Levinton, 2012; Lorenzi, Sella, Schleicherová, & Ramella, 2005; Santi, Picchi, & Lorenzi, 2018; Schleicherová et al., 2014).

In our study, we also primarily focus on plastic adjustments of SA in response to the social environment, and more specifically, to different mating group sizes. The effect of mating group size on SA can be viewed in terms of reaction norms, with a steep slope of the reaction norm representing greater plasticity in SA compared to a shallower slope. Moreover, the reaction norm can be thought of as an evolved trait that can vary between species, and it can serve as a useful tool for studying evolution of plasticity (Stearns, 1992).

To estimate SA in hermaphrodites, resource allocation toward the male and female function needs to be quantified, and such allocation can be considered to have both static and dynamic components. While the static component involves allocation toward the establishment and maintenance of reproductive organs, such as testes and ovaries, the dynamic component involves allocation toward the actual production of gametes (and possibly secretion products of other reproductive glands), leading to the output of the reproductive organs (Schärer, 2009). Studies generally tend to focus on estimating the relatively easy to measure static reproductive organs and then simply assume a tight correlation between the static measures (e.g., testis size) and the dynamic measures (e.g., sperm production rate). While this assumption is likely often warranted to some degree, it clearly does not always hold perfectly, with dynamic measures sometimes exceeding the output expected from static measures (e.g., Giannakara, Schärer, & Ramm, 2016; Lüpold, Linz, Rivers, Westneat, & Birkhead, 2009; Ramm & Stockley, 2010; Schärer & Vizoso, 2007). Thus, it is interesting to measure both static and dynamic components of SA (and the link between them). For time reasons, we do only the former for two of the species studied here and both the former and latter for a third species.

Furthermore, phenotypically plastic changes in sperm production rate could, at least in theory, affect sperm morphology, since sperm size might trade‐off with sperm quantity, in which case sperm competition might select more numerous and thus also smaller sperm (Immler et al., 2011; Kelly & Jennions, 2011; Snook, 2005). Additionally, an increase in sperm competition risk and/or intensity might under some conditions directly select for plasticity in sperm traits that increase the paternity share of a sperm donor (Pizzari & Parker, 2009; Snook, 2005). Several recent studies have shown phenotypic plasticity in sperm morphology and function (e.g., ascidians, Crean & Marshall, 2008; insects, Morrow, Leijon, & Meerupati, 2008; birds, Immler, Pryke, Birkhead, & Griffith, 2010; and flatworms, Janicke et al., 2016; but see Janicke & Schärer, 2010). Whether such sperm plasticity is widespread is unclear to date, which is why we explore this question in one of the species analyzed here (again for time reasons).

While SA plasticity in response to mating group size is widespread in hermaphroditic animals (reviewed in Schärer, 2009), the strength and nature of the plasticity vary both among and within species, with some studies showing plasticity only in the male function (Baeza, 2007; Hoch & Levinton, 2012; Janicke et al., 2013; Schärer & Janicke, 2009; Schärer & Ladurner, 2003; Winkler & Ramm, 2018) or the female function (Lorenzi et al., 2005; Schleicherová et al., 2014), while other studies show plasticity in either both functions (Janicke & Schärer, 2009, 2010) or no plasticity at all (Giannakara & Ramm, 2017). Although a part of this variation in these estimates of plasticity may reflect biases due to the ease with which male and female allocation can be measured in different study systems, it might also reflect different reproductive modes (with different postcopulatory processes, Schärer & Pen, 2013), species‐specific costs of plasticity (Schleicherová et al., 2014), or different evolutionary histories (Schleicherová, Sella, & Lorenzi, 2013). Comparative investigations of SA plasticity, within a phylogenetic framework, can be used to infer the extent to which SA plasticity evolves between related species and to identify the correlates that might be shaping its evolution. This can be done by comparing reaction norms of SA in response to mating group size across related species. Ideally, such comparative studies should be done in a consistent experimental paradigm, which is what we report on here.

The free‐living flatworm genus *Macrostomum* is an excellent model system for studying variation in SA plasticity, since reproductive organs can be measured accurately, repeatedly, and noninvasively in multiple species in the genus (e.g., Giannakara & Ramm, 2017; Schärer & Ladurner, 2003; Winkler & Ramm, 2018), allowing us to quantify and compare SA across closely related species. Earlier studies on the model organism *Macrostomum lignano* have shown that it has plastic SA, with SA increasing (i.e., becoming more male‐biased) with increasing group size (e.g., Schärer & Ladurner, 2003). In addition, both sperm production rate (Schärer & Vizoso, 2007) and sperm length (Janicke et al., 2016) increase with group size (though the latter only by about 3%; see also Janicke & Schärer, 2010).

In light of an emerging comparative context, we here studied SA plasticity in three *Macrostomum* species, namely *M*. *janickei*, *M. cliftonensis*, and *M. mirumnovem*, which are all close relatives of the established model species *M. lignano* (Schärer et al., 2019). To manipulate the mating group size, we raised worms in three different social group sizes—isolated, pairs, or octets—given that social group size is a good proxy for the mating group size, as previously shown for *M. lignano* (Janicke et al., 2013). Since manipulating the group size also changes the density at which the worms live—which could itself affect the reproductive allocation through various routes (e.g., resource competition or higher concentration of metabolites, as discussed by Schärer & Ladurner, 2003)—we explored this by manipulating density independently of the group size, by having two enclosure sizes (small and large) for each of the three group sizes. We expected that, if there was an effect of the density, both an increase in group size and a decrease in enclosure size will have similar effects on SA. In addition, in order to facilitate comparisons between species, we also determined standardized effect sizes on SA plasticity. And finally, we asked whether the treatments had any effect on sperm morphology (for *M. cliftonensis*) and we checked whether isolated worms were able to self‐fertilize.

## 2 MATERIALS AND METHODS

### 2.1 Study organisms

The three study species used here are all recently described free‐living flatworms of the genus *Macrostomum* (Macrostomorpha, Platyhelminthes), and they include *M. janickei* (see also Zadesenets, Schärer, & Rubtsov, 2017; Zadesenets et al., 2016), *M. cliftonensis*, and *M. mirumnovem* (Schärer et al., 2019). Briefly, the worms used in the experiments were from laboratory cultures that were established using individuals collected from Palavas‐les‐Flots, near Montpellier, France, for *M. janickei*; from Lake Clifton, South of Perth, Western Australia, for *M. cliftonensis*; and from Port Phillip Bay, Queenscliff, Victoria, Australia, for *M. mirumnovem* (Schärer et al., 2019). Moreover, these species are all close relatives of our main model species, *M. lignano*, with *M. janickei* being the closest, as shown by molecular phylogenetic analyses (Schärer et al., 2019), using *M. hystrix* as an out‐group species (see also Schärer, Littlewood, Waeschenbach, Yoshida, & Vizoso, 2011). All species are kept in mass cultures in the laboratory at 20°C in Petri dishes containing 32‰ artificial seawater (ASW) and fed with the diatom *Nitzschia curvilineata*.

### 2.2 Experimental design and objective

In our experiments, we raised worms in different group sizes (i.e., isolated, pairs or octets) and enclosure sizes (i.e., 24‐ or 6‐well tissue culture plates containing 1.5 or 6 ml of ASW, respectively) in all factorial combinations with ad libitum food and studied the effects of these treatments on reproductive allocation. The three treatments differ in their expected level of sperm competition, with the isolated treatment representing a nonreproductive state (or maximal LSC if worms self‐fertilize), with the paired treatment representing maximal LSC (or somewhat lower LSC if worms also self‐fertilize when in pairs, see below), and with the octet treatment representing low LSC (assuming worms engage in multiple mating; as shown for *M. lignano*, Janicke et al., 2013). Since the capability for self‐fertilization could potentially affect the level of LSC, we assessed for all species whether isolated worms could self‐fertilize. For all species, we measured body size (an estimate of overall size), ovary size (an estimate of female reproductive allocation), testis size (an estimate of male reproductive allocation), seminal vesicle size (an estimate of the amount of sperm available for transfer), and finally an estimate of SA (defined as testis size/(testis size + ovary size)). For *M. cliftonensis*, we further checked if the treatments had any effect on sperm production rate (by measuring the change in seminal vesicle size once a worm was isolated; cf. Schärer & Vizoso, 2007), and on different aspects of sperm morphology (cf. Janicke & Schärer, 2010).

Finally, using the obtained SA estimates, we compared the SA reaction norms between the different species. Specifically, we calculated standardized effect sizes to compare the effect of (a) the presence of mating partners (i.e., absent in isolated worms vs. present in worms in pairs and octets) and (b) the strength of sperm competition (i.e., low in pairs vs. high in octets) on SA plasticity in the different species.

### 2.3 Experimental procedures

To obtain hatchlings of the same age, we allowed adult worms to lay eggs for periods of 3, 2, and 3 days for *M. janickei*, *M. cliftonensis*, and *M. mirumnovem*, respectively (note that for *M. mirumnovem* we used three consecutive batches to obtain sufficient offspring). Six days after removing the adults, the resulting hatchlings of each species were randomly assigned to a specific group size and enclosure size. We replicated each factor combination at least 30 times (yielding *n* = 180 replicates per species at least). Worms were transferred to fresh culture plates every week and allowed to mature. Starting from days 44, 48, and 57 after egg laying for *M. janickei*, *M. cliftonensis*, and *M. mirumnovem*, respectively, we, over a period of 3 days, morphometrically measured a range of traits (see below) in one randomly chosen worm per replicate. For *M. mirumnovem*, we measured each of the three batches on its respective day 57, so as to measure all the worms at the same age. For *M. cliftonensis*, we, after measurement, isolated the worms for a period of 4 days to obtain an estimate of the sperm production rate and sperm morphology (see below). Then, starting from day 52, we took images of the body size, seminal vesicle size, and the sperm, again over a period of three days.

**Figure 1:**
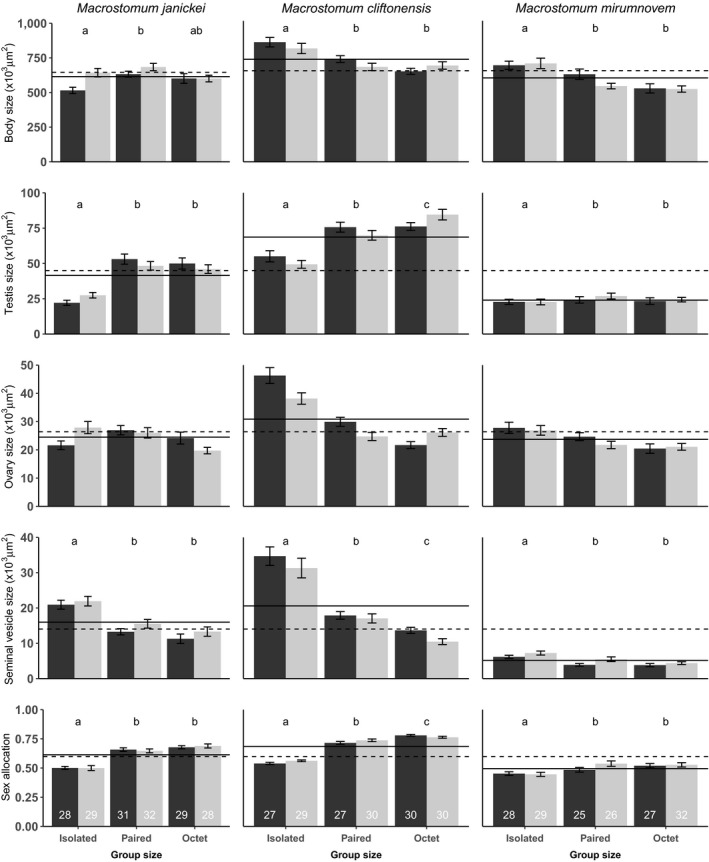
Effect of group size (*x*‐axis) and enclosure size (black bars: large; gray bars: small) on body size, testis size, ovary size, seminal vesicle size, and sex allocation in *Macrostomum janickei*, *Macrostomum cliftonensis*, and *Macrostomum mirumnovem*. Different letters denote significantly different group size effects inferred from Tukey HSD post hoc tests (not done for ovary size of *M. janickei* and *M. mirumnovem*, and done separately for small and large enclosures for ovary size of *M. cliftonensis*, see text). The solid line represents the mean for each species, and the dashed line represents the grand mean across all species. The plots show mean and standard error of raw data, but note that log‐transformed data were used for statistical analysis of testis and ovary size (in all species), body size (in *M. mirumnovem*), seminal vesicle size, and SA (in *M. cliftonensis*). Sample sizes for each treatment are given at the bottom

**Figure 2:**
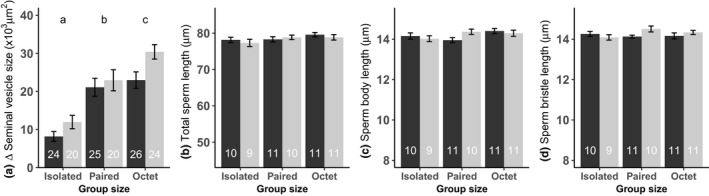
Effect of group size (*x*‐axis) and enclosure size (black bars, large; gray bars, small) on (a) increase in seminal vesicle size, (b) total sperm length, (c) sperm body length, and (d) sperm bristle length in *Macrostomum cliftonensis*. Different letters denote significantly different group size effects inferred from Tukey HSD post hoc tests. The plots show means and standard errors of raw data, though log‐transformed data were used for statistical analysis of total sperm length. Sample sizes for each treatment are given at the bottom

Across all species, some replicates had to be excluded due to the worm being malformed (*n* = 17), one worm missing in isolated (*n* = 7) or paired (*n* = 7) treatment replicates, or some mortality while handling worms (*n* = 12). Furthermore, for the sperm production rate estimates in *M. cliftonensis*, we lost an additional 34 worms, either due to a missing worm or some mortality while handling the worms. For the sperm morphology of *M. cliftonensis*, we only measured a subset of the worms for logistic reasons, chosen randomly such that all treatments were represented and culture plate effects avoided (see Figures 1 and 2 for final sample sizes).

### 2.4 Morphometry

For morphometry, we anaesthetized worms using a 7:5 mixture of 71.4 g/L MgCl_2_ solution and ASW and dorsoventrally squeezed them between a glass slide and a haemocytometer cover glass using standardized spacers (i.e., 40 µm for *M. janickei* and *M. cliftonensis*, and 45 µm for *M. mirumnovem* due to its somewhat larger body size; cf. Schärer & Ladurner, 2003). We then took images of the worms under a DM 2500 microscope (Leica Microsystems) using a digital camera (DFK41BF02; The Imaging Source) connected to a computer running BTV Pro 6.0b7 (Ben Software). The body size was imaged at 40×, while the testis, ovary, and seminal vesicle were imaged at 400× magnification. We analyzed these images using ImageJ (http://imagej.nih.gov/ij/) to obtain measures of body size, testis size (sum of both testes), ovary size (sum of both ovaries), and seminal vesicle size.

For each species, we obtained estimates of the repeatability of the above morphometric measurements, as given in Table S1, by imaging and measuring the same set of ~30 worms twice—randomly chosen from across all treatments—and then estimating the intraclass correlation coefficient (*r*_I_) using the variance components from a one‐way ANOVA with the R package “ICC” (Wolak, Fairbairn, & Paulsen, 2012).

For *M. cliftonensis*, we further estimated the sperm production rate, by measuring the increase in the seminal vesicle size in isolation between days 48 and 52 (see also Schärer & Vizoso, 2007). Moreover, we measured the sperm morphological traits of *M. cliftonensis* on days 52–55 using a procedure described in detail elsewhere (Janicke & Schärer, 2010). In brief, the tail plate of the worm was amputated with a scalpel and ruptured by transferring it in 1 µl of 32‰ ASW onto a glass slide and then covered with a coverslip (21 × 26 mm), thus rupturing the seminal vesicle and causing sperm to flow out where they could be imaged and measured approximately in a 2D plane. Similar to *M. lignano*, the sperm of *M. cliftonensis* has a complex design with a rapidly undulating anterior extension (termed feeler), followed by the sperm body, the sperm shaft, and the sperm brush at the end. In addition, there is a pair of stiff lateral bristles—anchored at the junction of the sperm body and shaft—which is hypothesized to represent a male persistence trait that allows the sperm to remain anchored in the female genitalia (see also Janicke et al., 2016; Schärer et al., 2011; Vizoso, Rieger, & Schärer, 2010). We imaged the sperm at 1,000× as above and measured the total sperm length (sum of length of sperm body and sperm shaft), sperm body length, and sperm bristle length of 10 sperm per individual (Janicke & Schärer, 2010), using the mean trait values per individual in the subsequent analyses.

### 2.5 Self‐fertilization

We assessed if the species can self‐fertilize by looking for offspring in the wells where the worms were being kept before being measured. We did this 10 days after the adults had been removed in order to give the eggs sufficient time to hatch. If a species could self‐fertilize, we expected the isolated treatments to also contain hatchlings.

### 2.6 Statistical analyses

To evaluate whether group size and enclosure size had any effect on body size, we calculated a two‐way (3 × 2) ANOVA with group size, enclosure size, and their interaction as fixed factors (and for *M. mirumnovem*, we also included batch number as a fixed factor). Furthermore, for testis, ovary, and seminal vesicle size we calculated the corresponding ANCOVAs, including body size as a covariate (since these traits were correlated with body size). In contrast, SA and the different sperm traits (i.e., total sperm length, sperm body length, and sperm bristle length) were analyzed without that covariate, since SA already represents a relative measure and the sperm traits were not found to be correlated with body size. To assess effects on sperm production rate in *M. cliftonensis*, we calculated the residuals of a linear regression of seminal vesicle size on the corresponding body size for days 48 and 52 (as estimates for the relative seminal vesicle size on that day), and then used the increase in these residuals (i.e., day 53 minus day 48) as the dependent variable, and performed the same two‐way ANOVA as above.

For all reproductive parameters for which the analyses suggested a significant group size effect, we calculated post hoc Tukey HSD tests to determine which levels differed significantly from each other. The data were graphically checked for the assumptions of parametric test statistics and transformed where needed, using log‐transformation for testis and ovary size (in all species); body size (in *M. mirumnovem*); and total sperm length, seminal vesicle size, and SA (in *M. cliftonensis*).

Finally, in order to facilitate comparisons of the level of SA plasticity in different *Macrostomum* species, we calculated standardized effect sizes using Cohen's *d* (Cohen, 1988) and confidence intervals (Howell, 2011) with the R package “effsize” (Torchiano, 2017). Specifically, in order to assess the effect of the presence of mating partners on SA we compared SA in isolated worms with that in pairs and octets (since worms in both pairs and octets have mating partners). Additionally, we assessed the effect of the strength of sperm competition by comparing SA in pairs with that in octets, with pairs representing high LSC and octets representing low LSC. All statistical analyses on our data were carried out using R, version 3.4.1 (R Core Team, 2017).

### 2.7 Prediction for SA response to group size

We expect that, with an increase in group size, sperm competition increases (and LSC decreases), as a result of which worms either mate more often or transfer more sperm per mating, leading to a higher sperm spending and a lower filling grade of the seminal vesicle. This then leads to a higher sperm production, in order to make up for the sperm depletion, which in turn favors an increased testis size and/or sperm production rate. Moreover, we expect that sperm production is likely not high enough to completely make up for sperm depletion, which should lead to a lower seminal vesicle size with increased group size, reflecting higher spending. Furthermore, under the assumption of a trade‐off between male and female reproductive allocation, we might expect a corresponding decrease in the ovary size as a result of the predicted increase in testis size, and thus an increasingly male‐biased SA as group size increases (although this SA trade‐off can be difficult to observe, cf. Picchi & Lorenzi, 2019; Schärer, 2009; Schärer, Sandner, & Michiels, 2005). Finally, we explore whether an increase in group size affects sperm morphology, either leading to larger sperm, since a large size could be advantageous in the context of high sperm competition, or potentially smaller sperm, since numerical competition might become more important as group size increases.

## 3 RESULTS

As we show in the following, all the studied *Macrostomum* species show phenotypic plasticity in SA, although they clearly do so to varying degrees. We first present the results for sex allocation plasticity in the three species separately, followed by the results about self‐fertilization and a comparison of the observed effect sizes.

### 3.1 Sex allocation plasticity in *M. janickei*

In *M. janickei*, body size was significantly affected by group size, with paired worms being significantly larger than isolated worms, while octet worms were intermediate in size and did not differ significantly from the other two groups (Table 1, Figure 1). In addition, worms in small enclosures were bigger compared to those in large enclosures, although care should be taken in interpreting these post hoc results, considering that the *p*‐value of the interaction term is relatively close to the significance level. In accordance with our expectations, testis and seminal vesicle size were strongly and significantly affected by group size, with isolated worms having significantly smaller testes and larger seminal vesicles compared to pairs and octets, while the enclosure size and interaction term had no significant effects, indicating that worms in larger groups invested more in the male function. The ovary area was not significantly affected by either group size, enclosure size, or their interaction. As seen for the testis size, SA was significantly affected by only the group size, with isolated worms being significantly different from pairs and octets. However, pairs and octets did not differ significantly in either testis size or SA, both of which we had expected based on predictions of SA theory.

**Table 1:** Effects of the fixed factors (group size, enclosure size, and their interaction) and the covariate (body size) on the dependent variables for *Macrostomum janickei*

Trait	Group size			Enclosure size			Interaction			Body size		
	*df*s	*F*	*p*	*df*s	*F*	*p*	*df*s	*F*	*p*	*df*s	*F*	*p*
Body size	2,171	4.61	**.01**	1,171	7.2	**.01**	2,171	2.73	.07	–	–	–
Testis size	2,170	63.2	**<.001**	1,170	1.36	.25	2,170	1.23	.3	1,170	96.9	**<.001**
Ovary size	2,170	1.95	.15	1,170	0.57	.45	2,170	1.19	.31	1,170	33.5	**<.001**
Seminal vesicle size	2,170	30.2	**<.001**	1,170	2.31	.13	2,170	0.25	.78	1,170	0.22	.64
SA	2,171	70.4	**<.001**	1,171	0.001	.97	2,171	0.24	.79	–	–	–

Note

Significant *p*‐values are highlighted in bold.

Results of ANOVA (body size and SA) and ANCOVA (all others) are shown.

### 3.2 Sex allocation and sperm morphology plasticity in *M. cliftonensis*

In *M. cliftonensis*, body size was significantly affected only by the group size, with isolated worms being significantly larger than worms in pairs and octets (Table 2, Figure 1). As predicted by SA theory, testis size increased significantly with group size, here differing significantly also between pairs and octets. The ovary size was significantly affected by both the group size and the interaction term, making it somewhat difficult to interpret the results biologically. We therefore performed the Tukey HSD post hoc tests separately for the small enclosures (isolated vs. pair, *p* = .01; pair vs. octet, *p* = .98; isolated vs. octet, *p* = .07) and large enclosures (isolated vs. pair, *p* = .01; pair vs. octet, *p* = .04; and isolated vs. octet, *p* < .001). In general, investment into ovaries was higher in smaller groups and larger enclosures, suggesting a trade‐off between the male and female allocation. Both group size and enclosure size had significant effects on seminal vesicle size (with the effect of the former being greater than that of the latter), with worms in larger groups and smaller enclosures having smaller seminal vesicles. And matching our expectation that increased sperm competition favors increased sperm production, sperm production rate (the increase in seminal vesicle size during the four days of isolation) was also significantly higher in larger groups, although care should be taken in interpreting these post hoc results, as the p‐value of the interaction term is relatively close to the significance level (Figure 2a). And finally, SA was affected by group size, with worms in larger groups having a higher SA in general, though the post hoc tests should be interpreted with some caution considering the marginally significant interaction term, which may stem from the above‐mentioned interaction term for ovary size (Table 2, Figure 1). None of the sperm morphological traits in *M. cliftonensis* were significantly affected by the group size, enclosure size, or their interaction (Table 2, Figure 2b–d), suggesting that phenotypic plasticity in testis size and sperm morphology do not correlate.

**Table 2:** Effects of the fixed factors (group size, enclosure size, and their interaction) and the covariate (body size) on the dependent variables for *Macrostomum cliftonensis*

Trait	Group size			Enclosure size			Interaction			Body size		
	*df*s	*F*	*p*	*df*s	*F*	*p*	*df*s	*F*	*p*	*df*s	*F*	*p*
Body size	2,167	18.15	**<.001**	1,167	0.62	.43	2,167	1.79	.17	–	–	–
Testis size	2,166	143.55	**<.001**	1,166	0.03	.85	2,166	0.82	.44	1,166	31.77	**<.001**
Ovary size	2,166	27.33	**<.001**	1,166	1.15	0.28	2,166	5.99	**.003**	1,166	204.62	**<.001**
Seminal vesicle size	2,166	45.41	**<.001**	1,166	5.03	**.03**	2,166	1.38	.26	1,166	36.52	**<.001**
Increase in seminal vesicle size	2,133	78.08	**<.001**	1,133	2.8	.1	2,133	2.63	.08	–	–	–
SA	2,167	306.47	**<.001**	1,167	2.02	.16	2,167	2.81	.06	–	–	–
Total sperm length	2,56	1.95	.15	1,56	0.31	.58	2,56	0.56	.57	–	–	–
Sperm body length	2,56	1.9	.16	1,56	0.29	.6	2,56	2.48	.09	–	–	–
Sperm bristle length	2,56	0.55	.58	1,56	1.87	.18	2,56	2.47	.09	–	–	–

Note

Significant *p*‐values are highlighted in bold.

Results of ANOVA (body size and SA) and ANCOVA (all others) are shown.

### 3.3 Sex allocation plasticity in *M. mirumnovem*

Similar to the results in the previous species, body size in *M. mirumnovem* was significantly affected only by the group size, with isolated worms being larger than worms in pairs and octets (Table 3, Figure 1). Again, and in agreement with our expectations, group size significantly affected testis size, with isolated worms having smaller testes than worms in pairs and octets, while pairs and octets did not differ significantly, though the interaction was relatively close to the significance level, so the post hoc results should be interpreted with some caution. The batch number also significantly affected testis size, with worms from batch 3 having significantly smaller testis (Tukey HSD test: Batch 1 vs. 2, *p* = .28; Batch 2 vs. 3, *p* = .0008; and Batch 3 vs. 1, *p* < .0001). Isolated worms had larger seminal vesicles than the other two treatments, in accordance with our expectations; however, pairs and octets did not differ significantly in this parameter. Moreover, enclosure size also had a significant effect, with worms in larger enclosures having smaller seminal vesicles, which was contrary to our expectations, since, if anything, we expected worms in smaller enclosures to donate more sperm. The ovary size was not significantly affected by any of the factors. Note that although a visual inspection appears to suggest a trend in *M. mirumnovem* for ovary size decreasing with increasing group size (Figure 1), this effect was not significant when correcting for body size, which shows a similar pattern. Similar to the testis, SA was significantly affected by group size and batch number, with isolated worms being significantly different from pairs and octets and batch 3 having significantly lower SA (Tukey HSD test: Batch 1 vs. 2, *p* = .19; Batch 2 vs. 3, *p* = .003; and Batch 3 vs. 1, *p* < .0001, Table 3). Worms from pairs and octets also did not differ significantly in SA.

**Table 3:** Effects of the fixed factors (group size, enclosure size, and their interaction) and the covariate (body size) on the dependent variables for *Macrostomum mirumnovem*. Note that we here also included the batch effect as a fixed factor

Trait	Group size			Enclosure size			Interaction			Batch effect			Body size		
	*df*s	*F*	*p*	*df*s	*F*	*p*	*df*s	*F*	*p*	*df*s	*F*	*p*	*df*s	*F*	*p*
Body size	2,158	17.33	**<.001**	1,158	0.59	.44	2,158	0.98	.38	2,158	1.7	.19	–	–	–
Testis size	2,157	11.95	**<.001**	1,157	2.72	.1	2,157	2.71	.07	2,157	12.36	**<.001**	1,157	69.23	**<.001**
Ovary size	2,157	0.28	.76	1,157	0.08	.78	2,157	0.82	.44	2,157	0.38	.69	1,157	227	**<.001**
Seminal vesicle size	2,157	8.08	**<.001**	1,157	9.16	**.003**	2,157	1.17	.31	2,157	1.56	.21	1,157	27.8	**<.001**
SA	2,158	10.1	**<.001**	1,158	1.59	.21	2,158	1.71	.19	2,158	11.02	**<.001**	–	–	–

Note

Significant *p*‐values are highlighted in bold.

Results of ANOVA (body size and SA) and ANCOVA (all others) are shown.

### 3.4 Self‐fertilization

Only *M. mirumnovem* was found to self‐fertilize during our observation period, with offspring being produced in 17 out of the 56 isolated replicates, while in a comparable number of isolated replicates the other two species never had any offspring (Table S2). Conversely, we found offspring in nearly all replicates containing pairs and in all replicates containing octets, irrespective of the species (Table S2), clearly suggesting that our holding conditions were generally favorable for reproduction.

### 3.5 Effect of presence of mating partner and local sperm competition

Overall, the presence of mating partners had a larger effect on SA than the strength of sperm competition in all the studied species, and of these species, *M. cliftonensis* was the most plastic, followed by *M. janickei* and *M. mirumnovem* (Table 4). This implies that the shift in our estimate of SA from the isolated to the pair/octet treatment is of a higher magnitude compared to the increase from pairs to octets.

**Table 4:** Standardized effect sizes (Cohen's *d*) for the effect of the presence of mating partners and sperm competition on SA

Species	Mating partner	Sperm competition
*Macrostomum janickei*	1.88 (1.51 to 2.26)	0.36 (0 to 0.72)
*Macrostomum cliftonensis*	3.73 (3.22 to 4.24)	0.82 (0.44 to 1.2)
*Macrostomum mirumnovem*	0.70 (0.36 to 1.03)	0.12 (−0.26 to 0.5)

Note

The 95% confidence intervals are given in brackets.

## 4 DISCUSSION

In agreement with earlier results obtained for the model organism *M. lignano* (Brauer et al., 2007; Janicke et al., 2013; Janicke & Schärer, 2009; Schärer & Ladurner, 2003; Schärer et al., 2005), we found that all the *Macrostomum* species analyzed here exhibit SA plasticity, with testes being more plastic than ovaries. Interestingly, despite the fact that *M. cliftonensis* is more distantly related to *M. lignano* than *M. janickei*, it showed plasticity patterns that were more similar to those of *M. lignano*. Specifically, SA increased significantly from pairs to octets—as previously seen in *M. lignano*—while the much more closely related *M. janickei* was less plastic, with pairs and octets having similar SA. This result suggests that SA plasticity can evolve readily, even in closely related species. Moreover, as in the earlier studies on *M. lignano*, it is group size rather than enclosure size that is primarily responsible for the observed shifts in SA, with the latter either not being significant or having smaller effects compared to the former on SA (as is evident from the much higher *F*‐values for group size compared to enclosure size across the species; Tables 1, 2, 3). This suggests that the observed shifts in SA are not simply caused by density effects, but that they largely result from changes in the number of interacting individuals (cf. Schärer & Ladurner, 2003). In the following, we discuss these results in some more detail.

In our study, we found that the response of body size to the treatments differed across species, with paired worms being larger than isolated worms for *M. janickei*, while for *M. cliftonensis* and *M. mirumnovem* the isolated worms were larger than paired and octet worms. While a potential reason for body size decreasing in larger groups could be resource competition, in our experiment the worms were fed ad libitum, which is why we consider this an unlikely explanation. Interestingly, also in earlier studies on *M. lignano* different trends for body size have been observed. Some studies showed that worms grow larger with increased group size (Brauer et al., 2007; Janicke et al., 2013), while another showed that isolated worms were smaller than worms in pairs (Schärer & Janicke, 2009), and still others showed that neither group nor enclosure size had an effect on body size (Janicke & Schärer, 2009; Schärer & Ladurner, 2003). While this could potentially complicate the interpretation of our results, since body size has been shown affecting SA in simultaneous hermaphrodites (Schärer, 2009; Vizoso & Schärer, 2007), in *M. lignano* SA (and particularly the testis size component of the SA estimate) was similarly plastic and increased with group size across all studies, irrespective of the trend for body size that was observed, pointing toward an effect of group size on SA that is largely independent of body size.

Furthermore, in our study testis size—our primary estimate for male reproductive allocation—increased with an increase in group size in a way similar to *M. lignano*, and in line with the prediction of SA theory (Charnov, 1982), though not to the same extent across the three species. In contrast, in all three species the ovary size did not appear to be as plastic as testis size. More specifically—and in contrast to the prediction of SA theory—there was no significant effect of group size on ovary size in *M. janickei* and *M. mirumnovem*. And while group size had a significant effect on ovary size for *M. cliftonensis*, the significant interaction term means that some care is needed to interpret that main effect. Briefly, although there seemed to be a trend for decrease in ovary size as the group size increased, isolated and paired treatment worms had a smaller ovary size in smaller enclosures, while octet treatment worms had a larger ovary size in smaller enclosures. One potential explanation for not observing a corresponding decrease in ovary size, in spite of the often marked increase in testis size, could be that ad libitum feeding masks the trade‐off between male and female reproduction function (cf. Schärer et al., 2005). Furthermore, male reproduction may not trade‐off with female reproduction, but with other life‐history traits (Schärer, 2009). However, there are several lines of evidence to suggest that a trade‐off between male and female allocation exists, at least in *M. lignano* (Janicke & Schärer, 2009; Schärer et al., 2005). Alternatively, ovary size may be an inferior proxy for measuring investment into the female function compared to testis size for the male function. This seems plausible, since at least part of the egg development, such as the addition of yolk and shell granules (Gremigni, 1988; Gremigni, Falleni, & Lucchesi, 1987), occurs outside the transparent portion of the ovary, which is what we generally use to estimate ovary size in *Macrostomum*.

We predicted that seminal vesicle size would decrease with increased group size (as worms likely mate more often and/or transfer more sperm per mating), and this is indeed what we found in all three species, though again to varying degrees. In all species, seminal vesicle size decreased significantly going from isolated worms to worms in pairs and octets, while only in *M. cliftonensis* did it further decrease significantly going from pairs to octets. Interestingly, these results mirror the observed differences in testis size between pairs and octets across the three species, which may support our notion that sperm production and testis size respond to the fill grade of the seminal vesicle. Moreover, in *M. cliftonensis* the enclosure size had a significant effect, with worms in smaller enclosures having smaller seminal vesicles. This could potentially be explained by considering that worms in smaller enclosures may encounter and mate with other individuals at a higher rate. Interestingly, we see the converse effect in *M. mirumnovem*, with worms in larger enclosures having smaller seminal vesicles, but we currently do not know of a plausible hypothesis for this observation.

As predicted by sex allocation theory (Charnov, 1982), SA increased with increasing group size, as worms start investing more in their male function and possibly somewhat less in their female function. However, the SA effect we see here likely stemmed primarily from the above‐mentioned variation in testis size, since the ovaries did not appear to be very plastic.

Generally, changes in SA should always be interpreted in the light of the changes observed in the different components used to estimate it (Schärer, 2009). In our study, we estimated SA as the proportion of testis size to overall gonad size (cf. Janicke et al., 2013; Janicke et al., 2016; Janicke & Schärer, 2009; Schärer & Janicke, 2009; Vellnow, Vizoso, Viktorin, & Schärer, 2017; Vizoso & Schärer, 2007), and we thus assume that testes and ovaries are comparably useful proxies for investment into male and female reproductive function. While estimating SA in this way allows us to compare relative differences in allocation to testes and ovaries between the different group sizes, it does not provide an absolute estimate of SA for a number of reasons. Firstly, while both testes and ovaries are involved in gamete production, the energetic expenditure per unit tissue could certainly differ between the two organs. Secondly, while testis size has been experimentally well‐verified to be quite a good proxy for male expenditure on sperm production rate (Giannakara et al., 2016; Schärer, Ladurner, & Rieger, 2004; Schärer & Vizoso, 2007), the support for ovary size being a good proxy for female reproductive investment is somewhat more limited (Schärer et al., 2005), as already mentioned above. And finally, there are certainly other components of both male and female reproduction, for example, sex‐specific behaviors, prostate glands, seminal fluids, copulatory organs, egg yolk production, eggshell glands, and eggshell material, that could potentially be plastic as well, and which we did not quantify here (Janicke et al., 2016; Patlar, Weber, & Ramm, 2019; Picchi & Lorenzi, 2019; Schärer, 2009; Schärer & Pen, 2013). Thus, our measures of male and female reproduction are not absolute, which is also reflected by the fact that our estimates of SA sometimes exceed 0.5 (Figure 1), which is not necessarily suggestive of a male‐biased SA. However, it appears likely that a greater sperm production will tend to go along with a greater expenditure on other male components (i.e., more ejaculate requires both more sperm and more seminal fluid), and similarly for egg production and female components (i.e., more eggs require both more oocytes and more yolk and shell material).

In one species, *M. cliftonensis*, we also looked at the sperm production rate, by measuring the increase in seminal vesicle size after isolating the worms, and found that it was significantly higher in larger group sizes. Thus, worms in larger groups produced sperm at a higher rate, likely to replenish the greater amounts of sperm that was being transferred under these conditions. Furthermore, despite the considerable plasticity in testis size and sperm productivity, the sperm morphology in *M. cliftonensis* did not show significant plasticity in response to variation in group size (or enclosure size). A recent study in *M. lignano* (with about 90 replicates per group size, Janicke et al., 2016) showed that worms raised in octets produce (about 3%) longer sperm than worms raised in pairs, while an earlier study in *M. lignano* ( *n* = 24 replicates per group size, Janicke & Schärer, 2010) found no significant plasticity in sperm morphology in response to group size, possibly since its statistical power was only expected to reliably detect effect sizes above 5%. Similar to the latter study, we here have a sample size of about 21 replicates per group size. Thus, it is possible that we may have failed to detect very small differences in sperm morphology between the treatments, though how biologically relevant such minor differences in sperm morphology are, is still an open question (see Janicke et al., 2016). In summary, the results of this study suggest that the genus has at most slight plasticity in sperm morphology in response to group size, particularly in comparison with the marked plasticity in a trait like testis size.

With respect to our estimates of standardized effect sizes, there was overall a stronger effect of the presence of a mating partner (i.e., isolated vs. pairs and octets) than the strength of sperm competition (i.e., pairs vs. octets) on SA for all three species (Table 4). This points toward relatively few resources being invested in sperm production (testes) in the absence of partners when no mating or fertilization can occur (unless by self‐fertilization), similar to *M. lignano* (Schärer & Janicke, 2009), while individuals upregulate sperm production in presence of partners either in response to the potential for mating or due to sperm expenditure occurring during mating. Another possibility could be that in the absence of partners there is little/no sperm expenditure such that the seminal vesicle fills up (Figure 1), which in turn may act as a signal to downregulate sperm production in isolated worms. Furthermore, at least in *M. lignano*, worms seem to be unable to distinguish between familiar and novel partners (Sandner & Schärer, 2010). This could mean that in pairs, even the single partner may be perceived as representing some sperm competition risk (though sperm competition is absent unless the species self‐fertilizes), leading to an increase in allocation into the male function.

Interestingly, we found that *M. mirumnovem* could self‐fertilize, since hatchlings were present in close to one third of the wells with isolated worms (who had grown up in isolation from hatchlings and were therefore virgins). The genus *Macrostomum* contains species exhibiting at least two different modes of reproduction (Schärer et al., 2011). The hypodermic mating syndrome involves presumably unilateral hypodermic mating, with sperm being injected via a sharp needle‐like male copulatory organ (stylet) into the parenchyma of the partner worm. In contrast, the reciprocal mating syndrome involves reciprocal copulation (e.g., *M. lignano*, see Schärer, Joss, & Sandner, 2004), during which both partners reciprocally transfer sperm into the female antrum (sperm receiving organ) of the partner. Both earlier reports of self‐fertilization in *Macrostomum* are in species that are hypodermically inseminating (e.g., *M. hystrix*, see Ramm, Vizoso, & Schärer, 2012; Ramm, Schlatter, Poirier, & Schärer, 2015; and *M. pusillum*, Giannakara & Ramm, 2017), with both species showing a similar needle‐like stylet morphology that potentially facilitates self‐fertilization by allowing self‐injection of sperm. In contrast, the current study documents the first occurrence of self‐fertilization in a reciprocally copulating species, *M. mirumnovem*, which has a large blunt‐ending stylet (Schärer et al., 2019), and for which we currently do not understand how selfing is actually achieved.

Being able to self‐fertilize would lead to high local sperm competition in isolated worms, thus favoring a lower allocation into the testis if self‐fertilization occurs frequently (Charlesworth & Charlesworth, 1981; Charnov, 1982; Schärer, 2009). In support of this, *M. mirumnovem* indeed has the lowest absolute testis and seminal vesicle size, as well as the lowest SA, compared to the other species (i.e., compare the species means to the grand mean across all species in Figure 1). A potential explanation for this could be that we used slightly thicker spacers for the standardized measurement of *M. mirumnovem* (i.e., 45 µm compared to 40 µm in *M. janickei* and *M. cliftonensis*), thus potentially leading to an underestimation of trait sizes. However, if that were the case, then all traits, including ovary size, should have been underestimated, but ovary size of *M. mirumnovem* was similar to that in the other species. Furthermore, the trend still holds even when looking at estimates of trait (testis, ovary, and seminal vesicle) size relative to body size for all species (data not shown). Thus, the low allocation into the male function may possibly be due to the fact that self‐fertilization in *M. mirumnovem* occurs with appreciable frequency. Interestingly, an earlier study in *M. pusillum*—a species that regularly self‐fertilizes—has shown that the species does not exhibit any plasticity in SA in response to group size (Giannakara & Ramm, 2017). In contrast, *M. hystrix*—a facultatively self‐fertilizing species—does adjust its SA depending on group size (Winkler & Ramm, 2018). This suggests that the degree of self‐fertilization could also influence sperm competition and therefore SA and its plasticity. In conclusion, to better understand the evolution of SA and the nature and extent of SA plasticity in the genus *Macrostomum*, more species need to be investigated, thus facilitating comparative studies of the evolution of SA and SA plasticity. This will allow us to understand how different factors, such as group size and the degree of self‐fertilization, can affect SA plasticity.

## CONFLICT OF INTERESTS

None declared.

## AUTHOR CONTRIBUTIONS

PS and LS designed the experiments. PS performed the experiments with some help from NV. PS analyzed the images. PS, NV, and LS statistically analyzed the data. PS and LS wrote the manuscript. All authors have read and approved the final version.

## Data Availability

All data in this manuscript will be deposited on Zenodo (https://doi.org/10.5281/zenodo.3333630).
