# Supplementary material for: Variation in sex allocation plasticity in three closely related flatworm species
Source: Ecol Evol. 2019 Aug 16;10(1):26–37. doi: 10.1002/ece3.5566 (PMC6972800; doi:10.1002/ece3.5566)
Supplement: Supplementary file 1 [file ECE3-10-26-s001.docx]

# Supplementary file to ‘Variation in sex allocation plasticity in three closely related flatworm species’

Table S1. Repeatability estimates (*r*_I_) of morphometric measurements for all species, with confidence intervals in brackets (all p < 0.05).

|  | *Macrostomum janickei* | | *Macrostomum* *cliftonensis* | | *Macrostomum* *mirumnovem* | |
| --- | --- | --- | --- | --- | --- | --- |
|  | F_34,35_ | *r*_I_ | F_30,31_ | *r*_I_ | F_26,27_ | *r*_I_ |
| Body size | 3.7 | 0.57 (0.34-0.79) | 2.9 | 0.48 (0.21-0.75) | 7.1 | 0.75 (0.59-0.92) |
| Testis size | 12.5 | 0.85 (0.76-0.94) | 7.5 | 0.76 (0.62-0.91) | 9 | 0.8 (0.66-0.94) |
| Ovary size | 10.9 | 0.83 (0.72-0.93) | 4.2 | 0.61 (0.39-0.84) | 5.8 | 0.7 (0.51-0.9) |
| Seminal vesicle size | 11.6 | 0.84 (0.74-0.94) | 15.2 | 0.88 (0.79-0.96) | 16 | 0.88 (0.8-0.97) |

Table S2. No. of replicates in which offspring were present or absent for each group size.

| Group size | *Macrostomum janickei* | | *Macrostomum* *cliftonensis* | | *Macrostomum* *mirumnovem* | |
| --- | --- | --- | --- | --- | --- | --- |
|  | Present | Absent | Present | Absent | Present | Absent |
| Isolated | 0 | 57 | 0 | 56 | 17 | 39 |
| Pair | 60 | 3 | 56 | 1 | 45 | 6 |
| Octet | 57 | 0 | 60 | 0 | 59 | 0 |
